# Sugar delivery at the tomato root and root galls after *Meloidogyne incognita* infestation

**DOI:** 10.1186/s12870-024-05157-7

**Published:** 2024-05-24

**Authors:** Lulu Sun, Liqiang Lian, Rui Yang, Tongtong Li, Minghui Yang, Wenchao Zhao, Huang Huang, Shaohui Wang

**Affiliations:** 1 Plant Science and Technology College, Beijing University of Agriculture, Beijing, 102206 China; 2 Beijing Key Laboratory for Agricultural Application and New Technique, Beijing University of Agriculture, Beijing, 102206 China

**Keywords:** Tomato, *Meloidogyne incognita*, Galls, Sugar unloading, Sugar delivery

## Abstract

Root-knot nematodes (RKNs) infect host plants and obtain nutrients such as sugars for their own development. Therefore, inhibiting the nutrient supply to RKNs may be an effective method for alleviating root-knot nematode disease. At present, the pathway by which sucrose is unloaded from the phloem cells to giant cells (GCs) in root galls and which genes related to sugar metabolism and transport play key roles in this process are unclear. In this study, we found that sugars could be unloaded into GCs only from neighboring phloem cells through the apoplastic pathway. With the development of galls, the contents of sucrose, fructose and glucose in the galls and adjacent tissue increased gradually. SUT1, SUT2, SWEET7a, STP10, SUS3 and SPS1 may provide sugar sources for GCs, while STP1, STP2 and STP12 may transport more sugar to phloem parenchyma cells. At the early stage of *Meloidogyne incognita* infestation, the sucrose content in tomato roots and leaves increased, while the glucose and fructose contents decreased. *SWEET7a*, *SPS1*, *INV-INH1*, *INV-INH2*, *SUS1* and *SUS3* likely play key roles in root sugar delivery. These results elucidated the pathway of sugar unloading in tomato galls and provided an important theoretical reference for eliminating the sugar source of RKNs and preventing root-knot nematode disease.

**Supplementary Information:**

The online version contains supplementary material available at 10.1186/s12870-024-05157-7.

## Introduction

Root-knot nematode disease is a common plant-specific parasitic disease caused by root-knot nematode (RKN) infestation of plant roots, which results in plant growth damage [1]. RKNs are devastating pests that can threaten dozens of plant species and cause crop yield losses [2]. At present, physical, chemical and biological means to treat root-knot nematode disease have poor and unstable effects or unfriendly to the environment. Additionally, plant cultivars containing the *mi* gene, which is resistant to RKNs, have lost their resistance effect at temperatures above 28° C [3]. Therefore, exploring more and better ways to treat root-knot nematode disease has become an important research direction.

After the RKN infects the plant root, several vascular parenchyma cells in the middle of the root are stimulated to expand, forming giant cells (GCs), and the root expand into the gall [4, 5]. A single root can be infected by multiple RKNs at the same time, leading to the formation of multiple galls. Galls are the “residence” of RKNs in plant roots, and RKNs rely on their stylets to absorb sugar, amino acids, water and other nutrient from GCs for their own growth and reproduction [6–8]. When the nutrient supply is sufficient, most nematodes develop into female worms and lay a large number of eggs, but when the supply of nutrients is insufficient, the development of RKNs stagnates, or most of them develop into male worms [9]. Surrounding a large amount of vascular tissue, GCs are similar to strong sink tissues, in which nutrients are obtained mainly from the unloading of surrounding vascular cells [10, 11].

Sucrose is the main form of phloem transport in most higher plants. The pathways associated with sucrose unloading from the phloem include the symplastic pathway, which is dependent on plasmodesma; the apoplastic pathway, which is dependent on sugar transporters; the mixed unloading pathway, which includes symplastic and apoplastic; and a switch unloading pathway: apoplasmic to symplasmic [12–14]. At present, the pathway through which sucrose is unloaded from phloem cells to GCs is not particularly clear [10]. An earlier study showed that when CF (carboxyfluorescein, a fluorescence tracer of phloem sap movement) was introduced from tomato leaves, CF signals could be found in the GCs of the root galls, indicating that the photoassimilates could be transported along the phloem to the root galls and finally unloaded into GCs through the symplastic unloading pathway [15]. A second study performed in Arabidopsis expressing GFP under control of *AtSUC2* promoter provides evidence that GCs are symplastically isolated [16]. However, another study on tobacco and Arabidopsis confirmed the existence of plasmodesmata (PD) between GCs and neighboring cells, whereas callose deposition along these PD was detected in tobacco roots but not in Arabidopsis, suggesting that GCs are symplastically isolated in tobacco but symplastically connected to neighboring cells through functional PD in Arabidopsis [17]. Another study demonstrated that symplastic connections exist between phloem and *Meloidogyne graminicola*-caused GCs in rice [18]. Based on the above research results, there were some contradictory conclusions in the above studies on the pathways involved in sugar unloading from the phloem cells to GCs in the gall, which might be caused by differences in the research methods, sampling times and species. Therefore, to clarify the source and unloading mechanism of sugar in GCs, further study is needed.

Studies have shown that after infestation, there are significant changes in sugar content and in the activities or gene expression levels of proteins related to sugar metabolism and transport, such as the sucrose transporter gene AtSUC1, which is significantly induced upon RKN infection [19]. RKN infestation caused a marked increase in total glucose-6-phosphate dehydrogenase (G6PDH) activity and protein abundance in wild-type Arabidopsis roots, and loss of G6PDH increased the susceptibility of *Arabidopsis thaliana* to RKN infestation [20]. Two months after the infestation of tomato plants with *Meloidogyne incognita*, the levels of sucrose, fructose, glucose and ribose were upregulated in the tomato stems [21]. A previous study suggested that soluble sugar (fructose, glucose and sucrose) content increased significantly in tomato leaves and roots during early infection by RKNs, and the expression levels of several sucrose transporters (SUTs/SUCs), Sugars Will Eventually be Exported Transporter (SWEETs), tonoplast monosaccharide transporter (TMTs), and vacuolar glucose transporter (VGT) gene family members were induced in the roots [22]. Another study also proved that SWEETs play important roles in plant and nematode interactions [23]. Do these changes mean that a large amount of sugar was unloaded from the phloem to the GCs after nematode infestation? Was there any difference between the early and late stages of RKN infection? What specific sugar metabolism and transport proteins play key roles in this process? The current knowledge does not give us a clear answer.

In this study, we used a combination of electron microscopy, CFDA tracing, sugar level analysis and RNA-seq analysis to study the structural changes in galls, the pathway involved in unloading sugars from the phloem to GCs, the changes in soluble sugars in the early and late stages of RKN inoculation, and the role of sugar metabolism and transport-related proteins in the formation of galls in tomato plants.

## Materials and methods

### Growth of plant materials and inoculation of RKNs

The seeds of tomato (*Solanum lycopersicum* cv. Castlemart) were supplied by Research Fellow Changbao Li (Beijing Vegetable Research Centre, Beijing Academy of Agriculture and Forestry Sciences) and germinated at 28℃ on moistened filter paper for 2–3 days, and then sown in seeding dishes filled with nursery substrates that were artificially mixed with a proportion of peat: vermiculite in a 2:1 ratio. The seedlings were grown in an artificial climate chamber (24℃–26℃/16℃–18℃,16 h light/8 h dark) until four true leaves stage. Eggs of *M. incognita* were obtained from the infected tomato roots according to previously described methods [24, 25], and incubated at 28℃ to hatch J2s. Each tomato plant was inoculated with approximately 500 J2s, and the galls were used for further experiments.

### Preparation and observation of paraffin sections of galls

Preparation of paraffin sections was performed as previously described [26]. Samples were collected at 0, 3, 4, 5, 6, 7, 14 and 21 days after inoculation (dpi). For 6, 7, 14 and 21 dpi, galls were collected. The samples were fixed in FAA solution (70% [v/v] ethyl alcohol, 5% [v/v] acetic acid, and 2% [v/v] formalin). Then the samples with the solution were pumped with a vacuum pump for 15 min and placed at room temperature overnight. Next, the samples were dehydrated in a graded ethanol series and transferred to xylene. Thereafter, the samples were infiltrated sequentially at a Paraplast: xylene volume ratio of 1:3, 1:1, and 3:1 for 4 h at 60 °C at each step. Then they were embedded in 100% Paraplast and cooled in the mold. Section 10 μm in thickness were cut using a microtome (Leica RM2255). Paraffin sections were stained with Fast green and then observed under an Olympus BX53 microscope.

### Transmission electron microscopy observations

Roots at 0, 3, 5 days and galls at 7, 14, 21 days after inoculation (dpi) were chosen to make ultrathin sections. The samples were fixed in 3% (w/v) glutaraldehyde (prepared with 100 mM phosphate buffer solution, pH 7.2) at room temperature for 6–9 h. Then the samples were washed, embedded, sliced and stained, as previously described [27]. Ultrathin sections were examined under a HITACHI-7650 transmission electron microscope.

### CFDA tracing assays

CFDA tracing assays were performed as previously described [13, 28] and there were some modifications. Carboxyfluorescein diacetate (CFDA, Sigma-Aldrich) solution was introduced into the phloem from the base of the tomato seedling (The nematodes had been inoculated for 3, 6 and 13 days the day before treatment, respectively) stem. Rub the stem surface to break the phloem (did not damage the xylem), then rinse the wound with 2.5 mM EDTA solution, and wrap cotton thread around the wound. The other end of the cotton thread was immersed in a tube with 100–300 µL 1 mg mL^− 1^ CFDA aqueous solution. Seedlings in the same stage introduced with water instead of CFDA solution were used as controls. After 24 h, the galls were picked and embedded in 6.5% low melting point agarose, and then cut into slices of 30 μm thickness using an oscillating microtome (Leica VT1000S). The slices were observed CF fluorescence at once using confocal laser scanning microscopy (CLSM, Leica Stellaris 5).

### Soluble sugars analyses

Roots and leaves samples were collected at 0, 12, 24, 36, 48 and 96 h after *M. incognita* inoculation, respectively. Galls and adjacent tissue were respectively collected at 7, 14 and 21 days after *M. incognita* inoculation. For soluble sugars analyses, samples were immediately drenched in liquid nitrogen.

The content of sucrose, fructose and glucose were measured by the ethanol extraction method [27, 29]. Soluble sugars were extracted from lyophilized tissues (0.2–0.5 g) in 5 ml of 80% (v/v) ethanol at 80 °C for 1 h. Then, samples were centrifuged at 4000 g for 10 min, and the supernatants were collected. The pellet was resuspended in 2–3 ml 80% (v/v) ethanol, and three rounds of extraction and centrifugation steps were performed. The three supernatants were combined and 0.1 g activated carbon was added in to adsorb the pigment and impurities, and then the filtrate was evaporated to dryness. The pellet was resuspended in 1 ml of deionized water, filtered through a 0.45 μm filter membrane and analyzed by high-performance liquid chromatography (HPLC, Agilent 1100). Three biological replicates were conducted for each sample.

### qRT-PCR assays

Root and leaf samples for early stage genes expression analysis were collected at 0, 12, 24, 36, 48 and 96 h after *M. incognita* inoculation, respectively. Galls and adjacent tissue were collected at 7, 14 and 21 days after *M. incognita* inoculation. Total RNA was extracted from 100 to 200 mg frozen roots or galls or leaves using Plant RNA Extract kit (DP432, Tiangen, Beijing, China) according to the manufacturer’s protocol, and reverse transcribed into cDNA using Reverse Transcription Kit (AT311–02, Transgen, Beijing, China).

Gene-specific primers and internal control (tubulin mRNA) primers (Table S1) were used to amplify PCR products on an ABI 7500 system (Bio-Rad). The reaction using SYBR Premix Ex Taq™ (Tli RNaseH Plus) (TaKaRa, Japan) according to the manufacturer’s protocol. Three biological replicates (samples from three individual plants) were performed and relative amounts of mRNA were calculated using the 2^−ΔΔCT^ method [30].

### RNA-seq analysis

Galls samples used for RNA-seq analysis were collected at 7, 14 and 21 days after *M. incognita* inoculation, and then divided into 7 groups according to the transverse diameter of the widest part of the gall. The gall formed after 7 days of inoculation was defined as RK1 for those with a transverse diameter of 0–0.5 mm, and RK2 for those with a transverse diameter of 0.5–1.0 mm. The gall formed after 14 days of inoculation was defined as RK3 for those with a transverse diameter of 1.0–1.5 mm and RK4 for those with a transverse diameters of 1.5–2.0 mm. The gall formed after 21 days of inoculation was defined as RK5 for those with a transverse diameter of 2.0–2.5 mm, and RK6 for those with a transverse diameter of 2.5–3.0 mm, and RK7 for those with a transverse diameter greater than 3.0 mm. To reduce interference information, ineligible galls from each plant were not sampled RNA-Seq.

RNA libraries were constructed and sequenced using Illumina HiSeq X-ten platform (Biomarker Biotechnology Co.). Each sample yielded more than 6 gigabytes of data. Clean data were aligned to the reference cucumber genome Chinese Long v2.0 via HISAT2. Quantification of gene expression levels were estimated by fragments per kilobase of transcript per million fragments mapped (FPKM). A corrected *P* value<0.01 and fold change>2 were set as the threshold for significantly differential expression. All genes in this RNA-Seq database related to sugar metabolism and transport were collected for comparative analysis.

### Statistical analyses

All measurements described were comprised of at least three biological replicates. Statistical analyses were performed using Microsoft Excel software. Student’s t test and Tukey’s Honestly Significant Difference test were used to evaluate the significant differences based on the IBM_SPSS_Statistics software.

## Results

### Formation of galls and vascularization of feeding sites

**Fig. 1:**
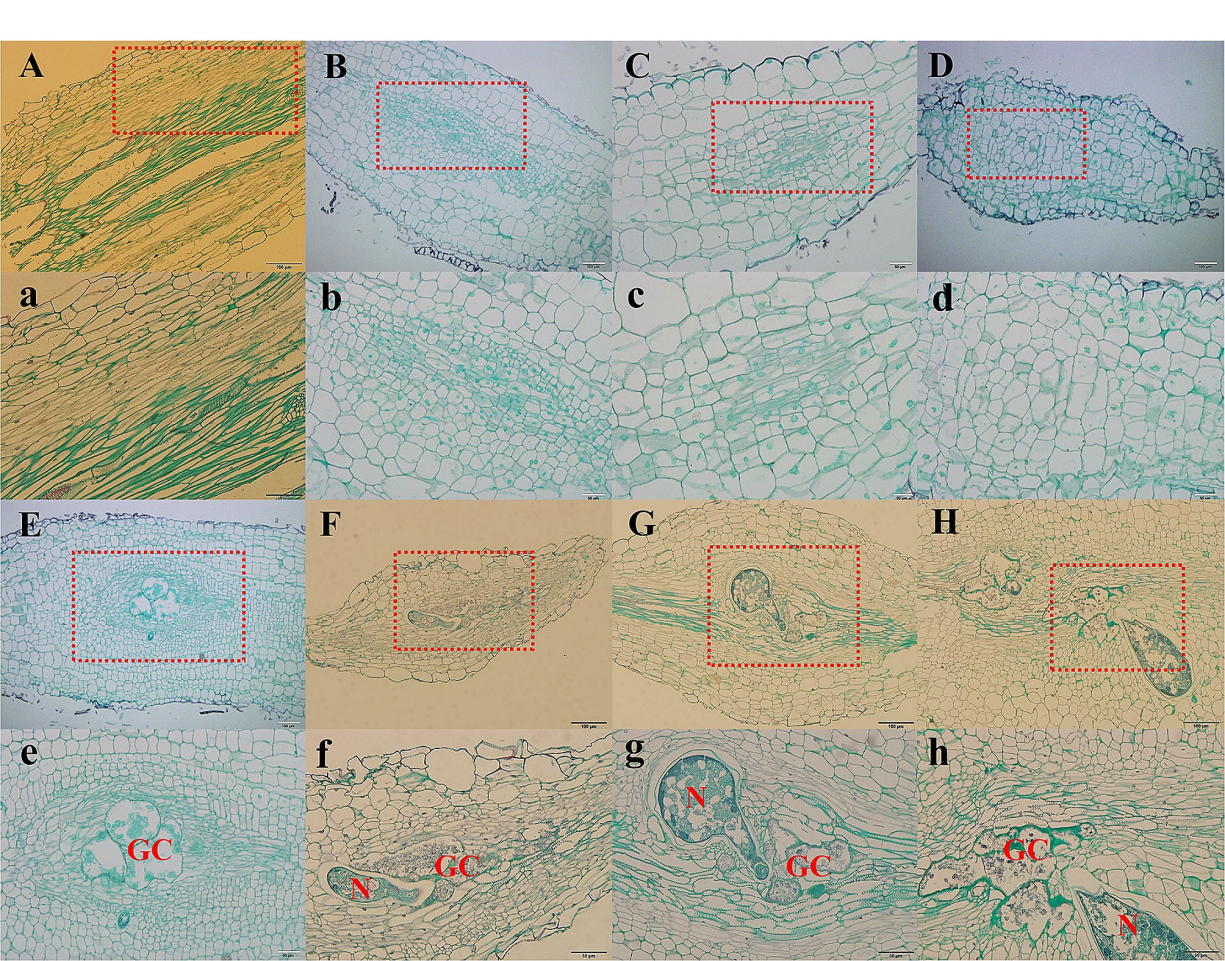
Longitudinal paraffin sections of roots or root galls at different stages after inoculation with *M. incognita*. (**A**) Uninoculated roots (0 dpi); (**B**) 3 dpi roots; (**C**) 4 dpi roots; (**D**) 5 dpi roots; (**E**) 6 dpi roots; (**F**) 7 dpi galls; (**G**) 14 dpi galls; (**H**) 21 dpi galls. a-h show enlargements of the images in the red boxes in A-H, respectively. Scale bars = 100 μm in (**A-H**) and 50 μm in (**a-h**). Abbreviations: GC, giant cell; N, root-knot nematode

The structure and vascular system of root galls are key to the study of nutrient delivery at root-knot nematode feeding sites in host roots. Xylem cells with threads, and phloem cells dyed green were clearly observed in the longitudinal sections of the 0 dpi roots (Fig. 1A and a). After 3–5 days of *M. incognita* inoculation, several vascular bundle parenchyma cells in the center of the roots were stimulated and gradually expanded (Fig. 1B-D and b-d). Visible galls could form approximately 6 days after nematode inoculation, and sometimes two or three adjacent galls joined together to form long and large galls (Fig. 1E-H and e-h). Structurally, the expansion of GCs in galls is also a process of vascular tissue proliferation and reconstruction. The original longitudinal vascular tissue was stretched from the middle to both sides with the expansion of GCs, and the large amount of new vascular tissue that formed by cell division gradually formed a dense reticular structure around the GCs. In addition, the GCs were irregular in shape and contained multiple nuclei and abundant contents. These 3–5 GCs were adjacent to each other; these GCs constitute the feeding region of the RKN stylets and provide nutrients for the growth and development of RKNs (Fig. 1E-H). Paraffin sections also showed many xylem cells with threaded inner walls and phloem cells dyed green surrounded the GCs. The green phloem cells in the 21 dpi galls had clear nuclei, but phloem cell types such as sieve element (SE), companion cell (CC), or phloem parenchyma cell (PP) could not be distinguished.

### Analysis of the pathway through which phloem nutrients are unloaded to GCs

**Fig. 2:**
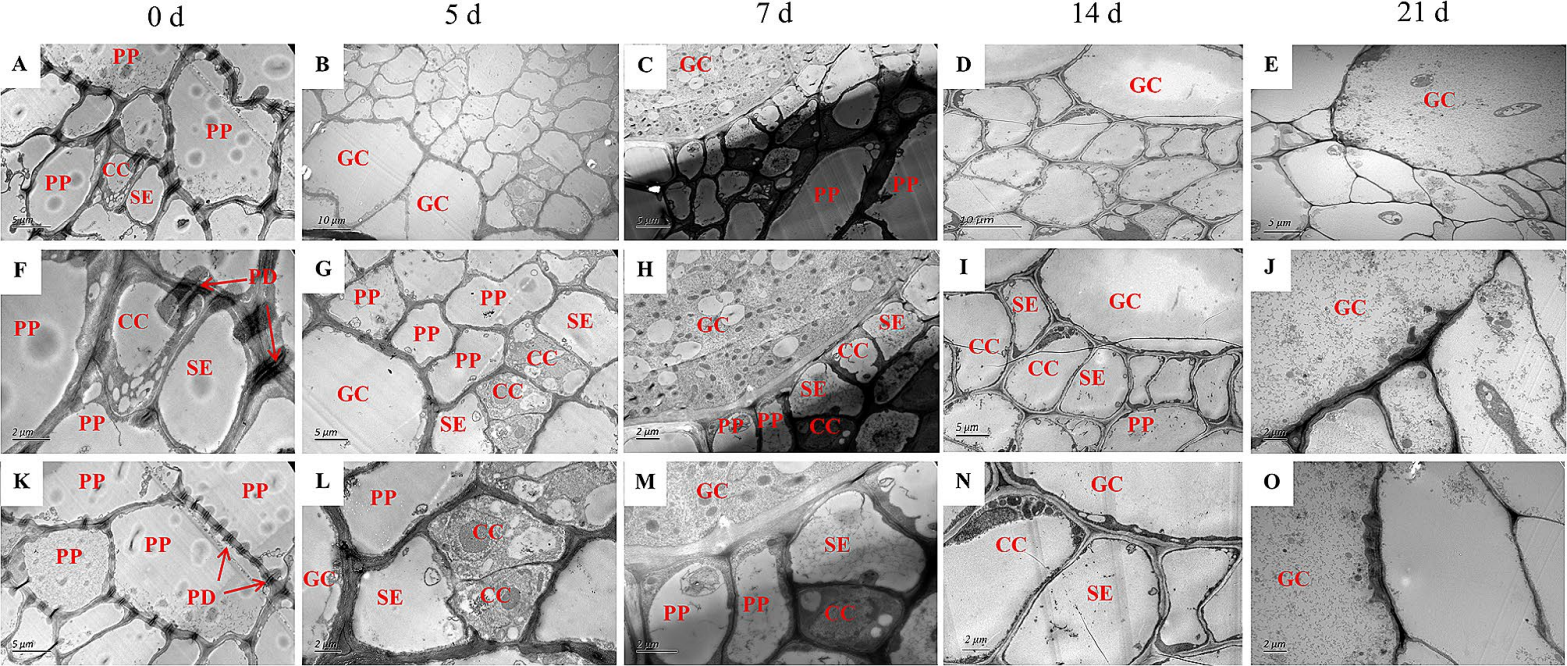
Ultrastructure of the GCs and surrounding vascular tissue cells in the roots or root galls at 5, 7, 14 and 21 days after RKN inoculation; the uninoculated roots served as a control (0 days). All the sections were cut transversely. **A-E**: Ultrastructure of the roots or galls. **F-J** Cell types of vascular tissue cells surrounding GCs. The SEs, CCs, PPs and GCs are labeled. **K-O**: The ultrastructure between GCs and SE/CCs and plasmodesmata are labeled with red arrows. Abbreviations: SE, sieve element; CC, companion cell; PP, phloem parenchyma cell; PD, plasmodesmata; GC, giant cell

RKNs extract nutrients such as sugars from GCs through their stylets through the unloading of peripheral phloem cells. To clarify the type of phloem cells surrounding the GCs and the pathway of nutrient unloading from the phloem cells to the GCs, transmission electron microscopy (TEM) was used to observe the ultrastructure of the GCs and the surrounding vascular tissue cells at 5, 7, 14 and 21 days after nematode inoculation, while the uninoculated root (0 days) served as a control. The uninfected roots clearly showed distinguishable SEs, CCs, PPs and a large number of plasmodesmata were found between PPs and SE/CCs (Fig. 2A, F, K). Plasmodesmata play pivotal roles in sucrose transfer between cells through the symplastic pathway [18]. At the early stage of the gall formation (5–14 days), the vascular tissue cells surrounding the GCs contained SEs, CCs and PPs (Fig. 2B-D, G-I); however, at 21 days, the only cell type around the GCs was SEs (Fig. 2E and J), presumably because the contents in the previous CCs disappeared and became SEs to facilitate rapid unloading of nutrients [16]. In addition, plasmodesmata were not found between GCs and surrounding vascular tissue cells (Fig. 2L-O), indicating that phloem cells unloaded nutrients to GCs through the apoplastic pathway. These results indicated that with the expansion of GCs, CCs become SEs in vascular cells, GCs become symplastic isolated from surrounding cells, and nutrients need to be transported to GCs through the apoplastic pathway.

**Fig. 3:**
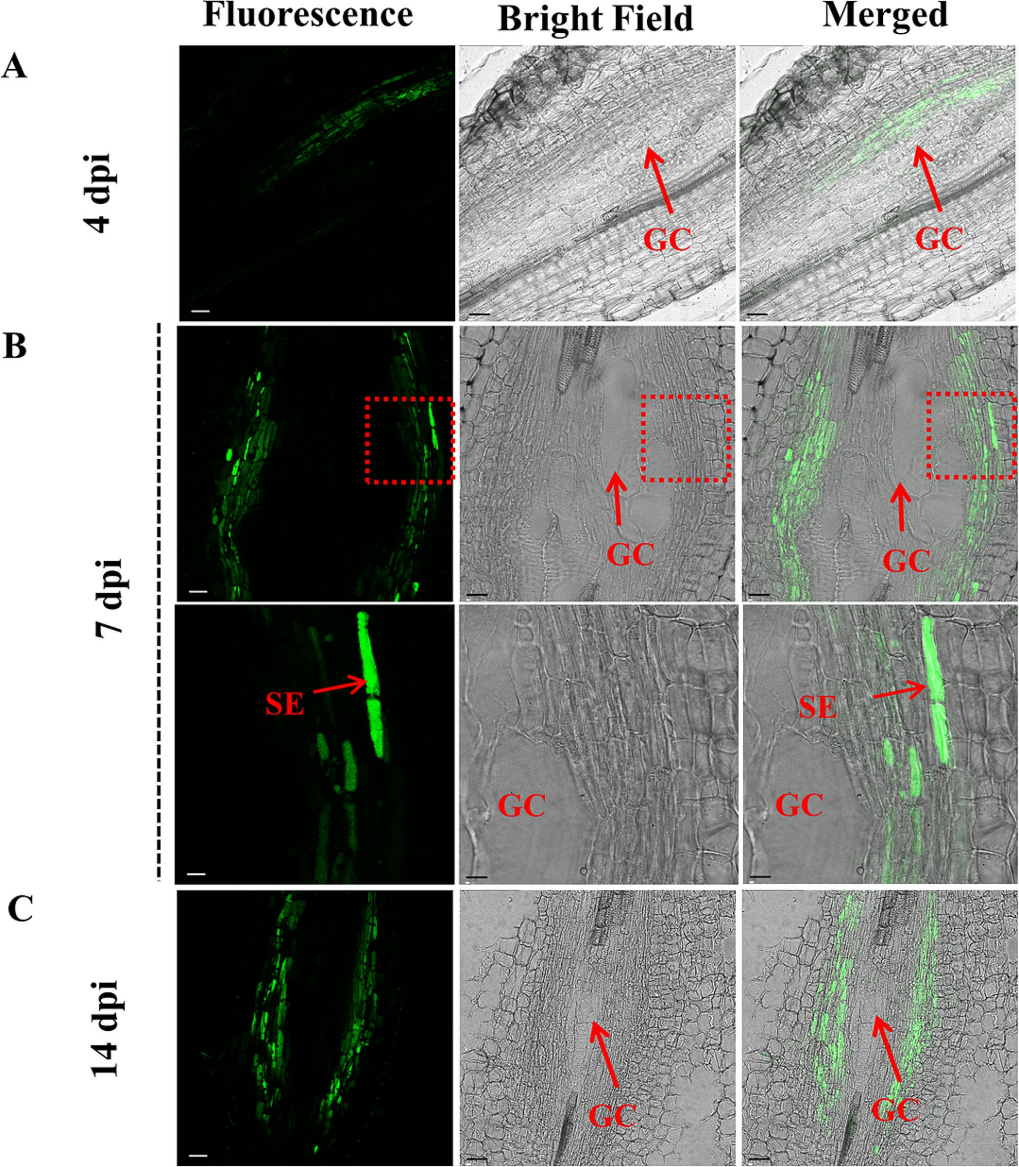
Confocal laser scanning microscopy (CLSM) imaging of carboxyfluorescein (CF) unloading during the development of root galls. The treated root or galls was sampled 24 h after CF tracing and cut into 30 μm thick slices using an oscillating microtome for CF fluorescence observation. **A-C** Fluorescence images of root or galls after inoculation with RKN at 4, 7 and 14 days. Scale bars = 20 μm in (**A** and **C**); scale bars = 15 μm (upper) and 50 μm (lower) in (**B)**. Abbreviations: GC, giant cell

We also used the CFDA fluorescent tracing method to study the unloading pathway of phloem nutrients to GCs. CFDA was introduced into the phloem sap from the base of the tomato stem and reached the gall following the sap. The results indicated that green fluorescence was always present around GCs in the roots or galls after 4, 7 and 14 days, and that green fluorescence did not enter GCs, suggesting that there were no plasmodesmata between GCs and surrounding cells and that nutrients could be transported only to GCs through the apoplastic pathway (Fig. 3; Fig. S1). This result was consistent with the TEM results.

### Analysis of soluble sugar content and sugar delivery mechanism in root galls

**Fig. 4:**
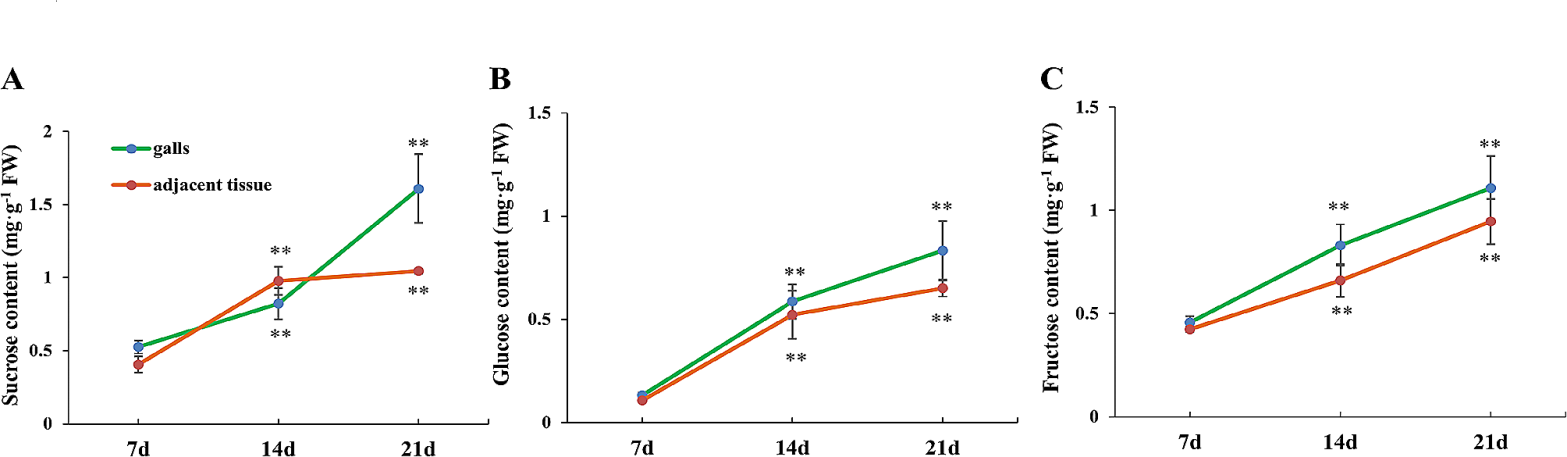
Soluble sugar content analysis of galls and adjacent tissue at 7, 14 and 21 days after RKN inoculation. The data are presented as the means ± SDs (*n* = 3) with units of mg/g fresh weight (FW). Student’s t test, *P < 0.05, **P < 0.01

To explore the mechanism of sugar transport from phloem cells to GCs, we first measured the soluble sugar content in the galls and the adjacent tissue at 7 d, 14 d and 21 d after RKN inoculation. The data showed that the sucrose, glucose, and fructose contents increased with the development of galls, both in the galls and adjacent tissue (Fig. 4).

**Fig. 5:**
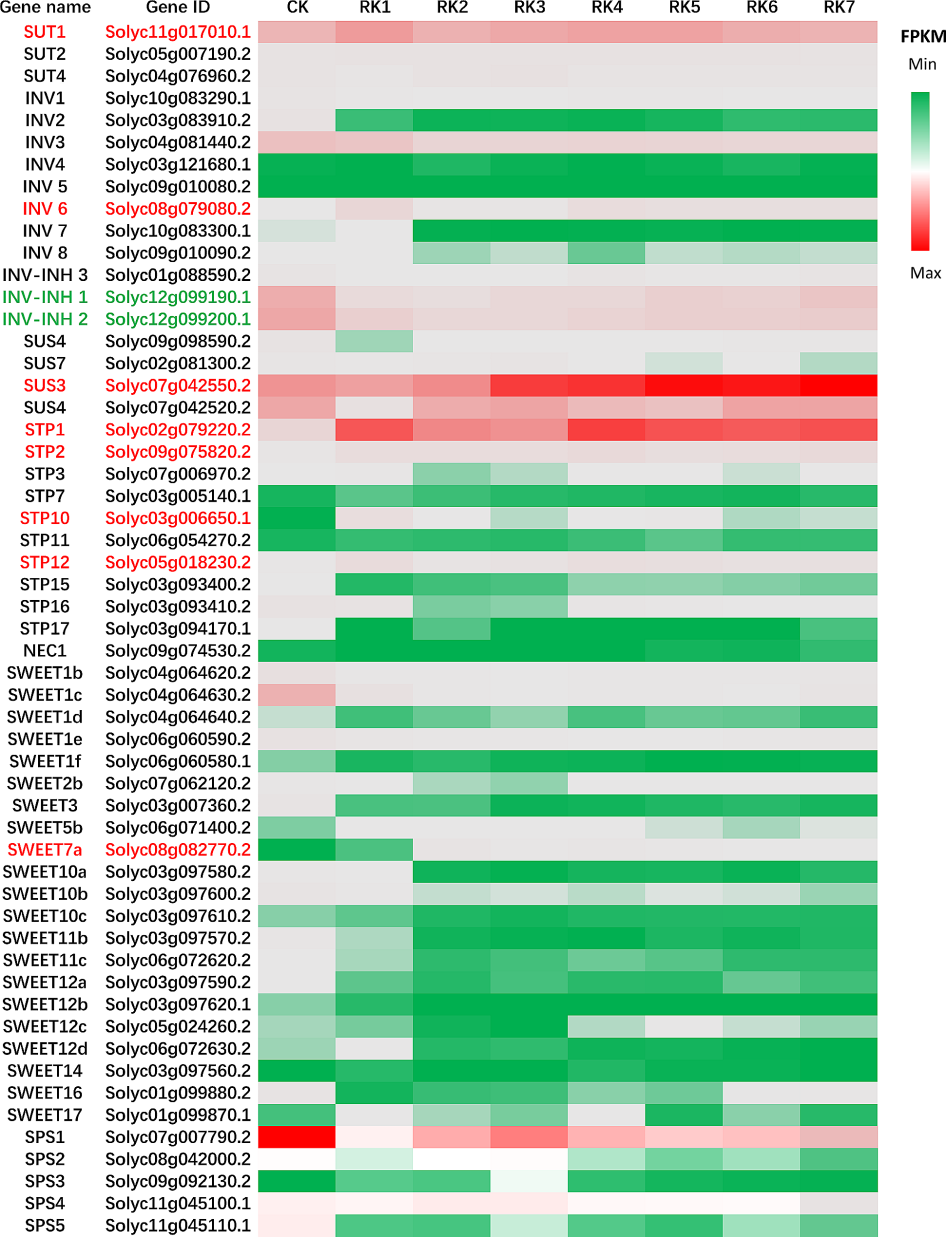
Sugar metabolism and transport genes expressed in roots of different sizes according to RNA-seq analysis. The gene IDs, names and FPKM values are shown in the picture. Abbreviations: RK, root knot (gall)

To further analyze which genes related to sugar metabolism and transport play key roles in the gall development and provide a reference for the subsequent use of these genes to obtain germplasm materials resistant to RKNs, different diameters of galls were used for RNA-seq analysis. RNA-seq data revealed that 55 genes related to sugar metabolism and transport were expressed in the galls. By classifying these genes according to function and analyzing their changes in expression in response to different sizes of galls. We found that the expression of a sucrose transporter gene *SUT1*, an invertase gene *INV6*, a sucrose synthase gene *SUS3*, four sugar transporter protein genes *STP1*, *STP2*, *STP10* and *STP12*, and a SWEET protein gene *SWEET7a* were significantly up-regulated, while the expression of two invertase inhibitor genes *INV-INH1* and *INV-INH2* were significantly down-regulated with the development of galls, indicating that these genes are most likely involved in sugar content changes in galls, which correlated with their functions (Fig. 5; Supplementary Table S2). SUT1 is responsible for the transmembrane transport of sucrose and has been demonstrated to be localized in SEs in sink tissues [31], suggesting a key role in phloem sucrose unloading. Sucrose is hydrolyzed to fructose and glucose by either invertase or SUS [32], and the activity of invertase can be inhibited by the inhibitor INV-INH [33]. STPs are generally considered to transport monosaccharides across membranes from the apoplast space to sink cells [34–37]. SWEET proteins can transport monosaccharides and/or disaccharides across membranes via a concentration gradient [38, 39]. SlSWEET7a was thought to transport mono- and disaccharides [40].

**Fig. 6:**
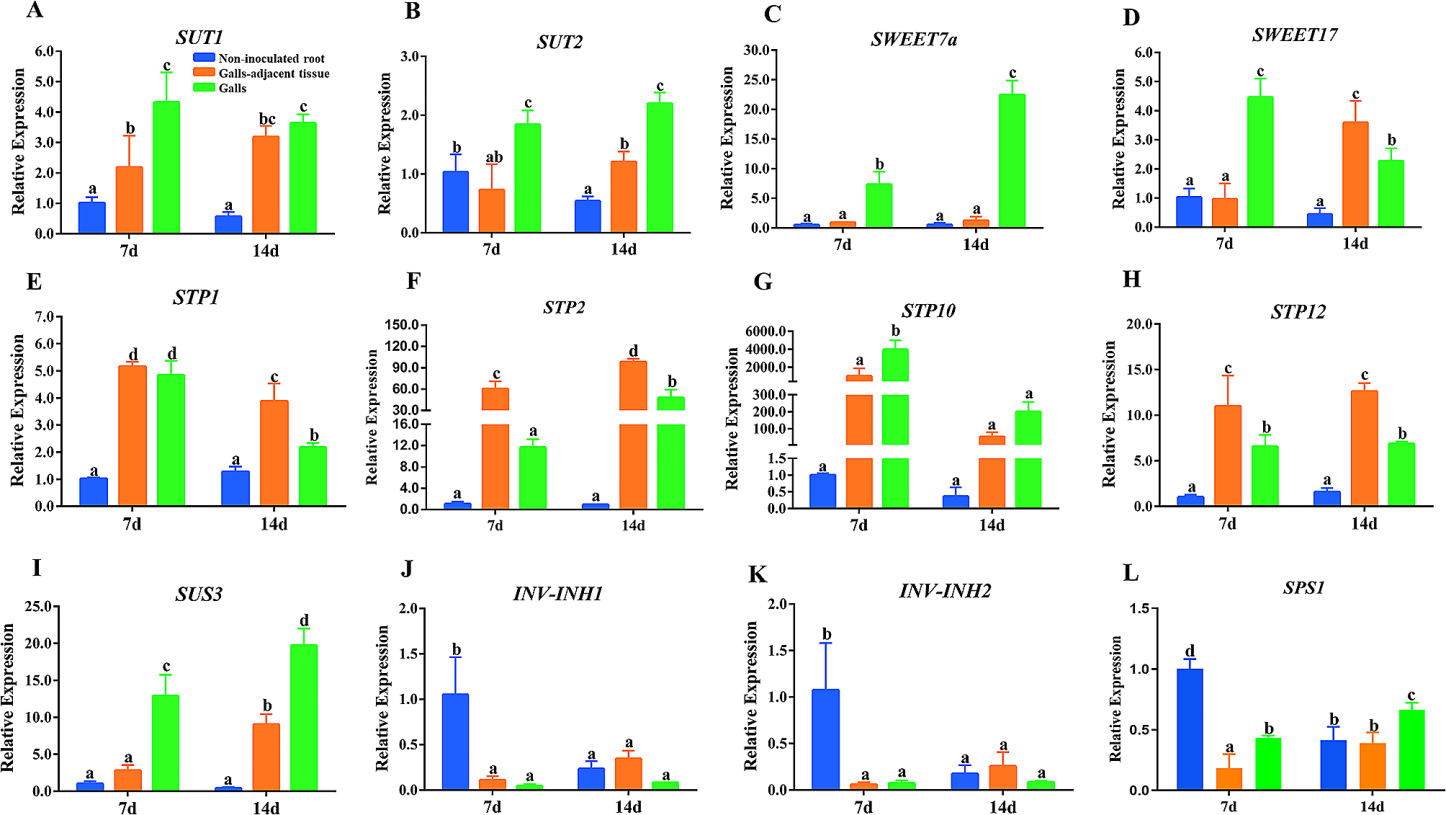
The mRNA levels of sugar metabolism and transport genes in non-inoculated root, galls-adjacent tissue and galls at 7 and 14 days after RKN inoculation. An ANOVA was performed using the Tukey honestly significant difference test, *n* = 3; the letters above the bars indicate significant differences (*P* < 0.05)

To further investigate the specific roles of these genes in the interaction between RKNs and host plants, galls and adjacent tissue at 7 d and 14 d after RKN inoculation were used to analyze the changes in the expression of these genes and several additional key genes involved in sugar metabolism. These sugar metabolism- and transport-related genes were upregulated, while the expression of *INV-INH1*, *INV-INH2* and *SPS1* was downregulated in the galls in response to RKN infection compared to that in the non-inoculated control root; these findings are closely related to the upregulation of soluble sugars in the galls (Fig. 6). The expression levels of *SUT1*, *SUT2*, *SWEET7a*, *STP10*, *SUS3* and *SPS1* in galls were greater than those in the adjacent tissue, suggesting that these proteins may provide sugar sources for GCs. However, the expression levels of *STP1*, *STP2* and *STP12* in the galls-adjacent tissue were greater than those in the same period, suggesting that these plants may transport more sugar to phloem parenchyma cells for root development than for the gall development. These results suggest that different sugar metabolism and transport genes play different roles in sugar delivery to galls.

### Changes in sugar metabolism at the early stage after *M. incognita* inoculation

**Fig. 7:**
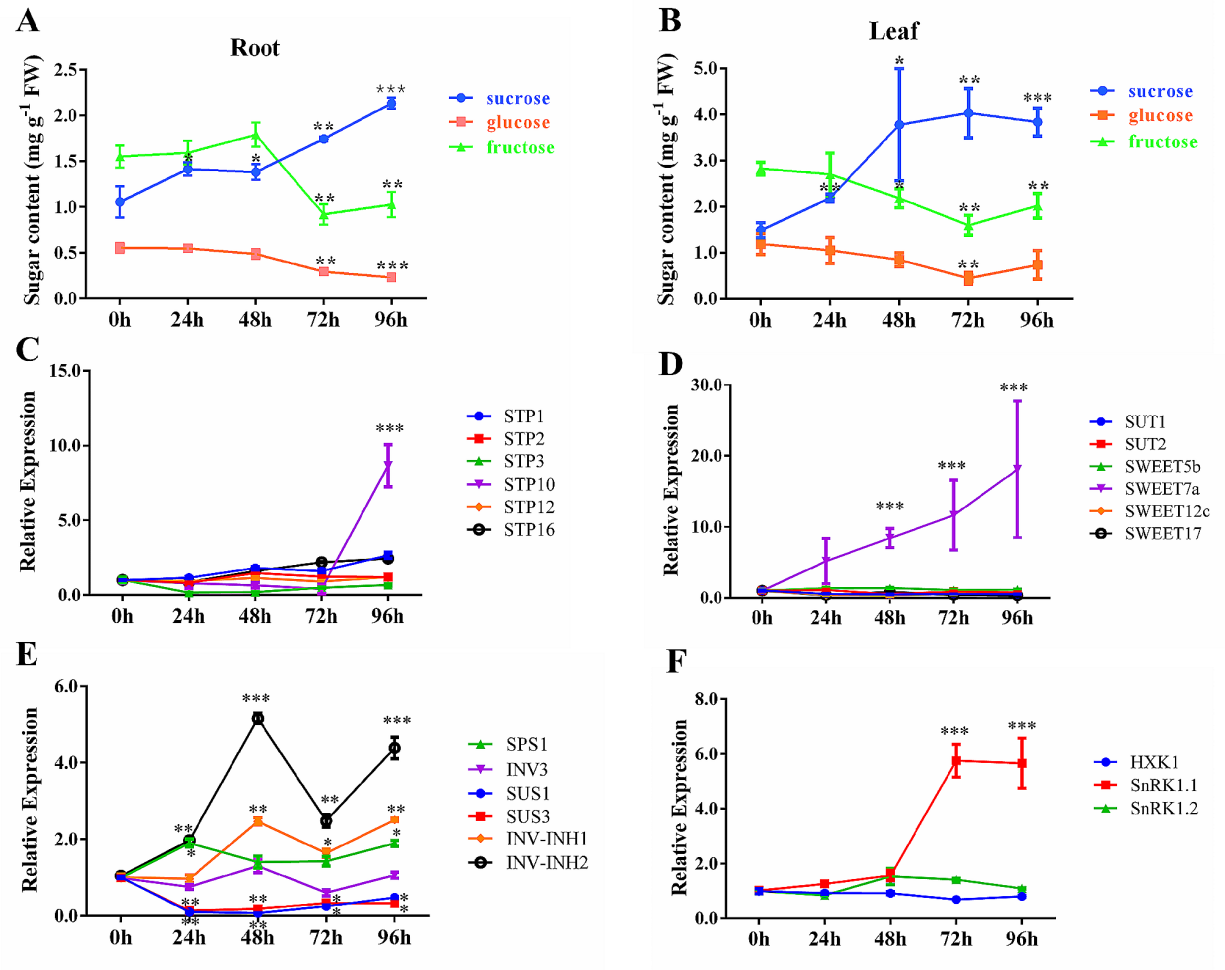
Analysis of soluble sugar content and gene expression in host roots and leaves at the early stage after RKN inoculation. Soluble sugar levels in the host roots (**A**) and leaves (**B**). The data are presented as the means ± SDs (*n* = 3) with units of mg/g fresh weight (FW). Student’s t test, *P < 0.05, **P < 0.01, ***P < 0.001. The mRNA levels of sugar transport protein genes (**C**), sucrose transporter and SWEET protein genes (**D**), enzyme-encoding genes related to sugar metabolism (**E**), and signal transduction pathway-related genes (**F**) in the roots were analyzed. The data are presented as the means ± SDs (*n* = 3). Student’s t test, *P < 0.05, **P < 0.01, ***P < 0.001

One study showed that RKNs could cause an increase in soluble sugar levels in plants at the initial stage of infection [22]. We examined the changes in soluble sugar content at the early stage (0–96 h after RKNs inoculation) of *M. incognita* infestation and found that the sucrose content in the roots gradually increased, but the glucose and fructose contents tended to decrease (Fig. 7A). Moreover, the changes in sucrose, glucose and fructose contents in the leaves were consistent with those in the roots during the same period (Fig. 7B).

To further determine which key genes involved in sugar metabolism and transport play roles during this stage, the expression levels of 17 related genes (based on published reports and online databases) were analyzed. Most of the genes did not change significantly during this process, except for *SWEET7a*, *STP10*, *SPS1*, *INV*-*INH1* and *INV-INH2*, whose expression levels were significantly upregulated, while *SUS1* and *SUS3* were significantly down-regulated. SWEET7a has been reported to transport sucrose and hexose [40]. STP10 can transport hexose into cytoplasm. SPS1 can promote sucrose synthesis. INV-INH1 and INV-INH2 are inhibitors of invertase that inhibit invertase activity and reduce the breakdown of sucrose. SUS1 and SUS3 contribute to the decomposition of sucrose. These results suggested that these genes may be related to the increase in sucrose content and the decrease in the sum of the fructose and glucose contents, and likely play key roles in root sugar delivery (Fig. 7C-E).

## Discussion

### The galls formation is a result of GCs growth and vascular bundle cell proliferation

In the roots of the host plant, RKNs induce highly specialized feeding sites (GCs) to optimize nutrient uptake [5, 8]. A previous study showed that the expansion of GCs disrupted the connectivity of the adjacent root phloem, and a large number of SEs were formed de novo and contained nuclei surrounding the GCs [16]. In our study, paraffin sections of roots or galls obtained at 3, 4, 5, 6, 7, 14 and 21 days after inoculation were collected, and the results suggested that the formation of vascular tissues around GCs was more consistent with the following hypothesis. Several parenchyma cells in the anchored area of tomato roots after RKN invasion become GCs that can provide nutrients for RKNs. GCs gradually compress the original vascular tissue from the middle, and a large number of new vascular cells differentiate into vascular tissues, forming a network structure around GCs, which may transport nutrients to GCs (Fig. 1).

Paraffin sections observation show that there were clear nuclei in the neighbor cells of GCs at the 21 dpi galls (Fig. 1E), but the phloem cell type could not be distinguished. TEM clearly revealed the cell types and connections between GCs and surrounding cells. In this study, we observed normal SEs, CCs and PPs around GCs at the early stage of the gall formation (5–14 days), although only PPs and SEs with nuclei were found at the later stage of the gall formation (21 days), which implied that CCs may gradually disappear or become SEs with the gall development (Fig. 2G-J). This result was consistent with previous findings that there were a large number of nucleated SEs around GCs in the later stage of Arabidopsis gall development [16]. It is not clear why the companion cells are absent [10], one guess is SEs in galls are nucleate and able to function without CCs, and these SEs with nuclei are heavily interconnected by PD that form a symplastic domain and allow for macromolecular trafficking [16].

### GCs were isolated from surrounding phloem cells via symplastic isolation

At present, the pathway through which carbohydrate is unloaded from the gall phloem cells to GCs has not been fully elucidated, and contradictory conclusions have been drawn from previous studies [10, 15–17]. A study showed that CF was applied to tomato leaves, 5 weeks after *M. incognita* inoculation, then the plants were incubated in the greenhouse for 1–3 days, and was sectioned from leaf to roots to determine dye movement. The results indicated the photoassimilates could be unloaded into GCs through the symplastic unloading pathway [15], despite the earlier study has shown that few plasmodesmatal connections have been found between giant transfer cells and neighbouring cells [41]. A second study performed in Arabidopsis proved that GCs are symplastically isolated. This study inoculated 4-week-old seedlings with *M. incognita* juveniles and performed microscopic observations at 18 dai [16]. . Another study on Arabidopsis confirmed PD were distributed along the entire cell wall of GCs and no callose deposition was detected, suggesting that GCs are symplastically connected to neighboring cells through functional PD. In this study younger Arabidopsis seedlings (12 days old) were inoculated and observed at 13 dai. In addition, this study also showed symplastically isolation of GCs from neighboring cells in tobacco [17]. These inconsistent conclusions in the above studies about the unloading pathway may be caused by differences in the research methods, sampling times and species. In this study, the roots and galls of tomato plants at different times after inoculation with RKNs were analyzed. TEM observation revealed that there was no plasmodesmata between GCs and surrounding cells at either the early or late stage of the gall development (Fig. 2L-O), and the results of the CFDA tracer experiment confirmed this conclusion (Fig. 3), indicating that sugars could be unloaded into GCs only through the apoplastic pathway.

### Sugar metabolism- and transport-related genes play important roles in the development of galls

Many studies have shown that RKNs can cause the soluble sugar content in roots to increase after infection [21, 22]. In the present study, after GCs formation, the levels of sucrose, fructose, and glucose increased with the development of galls and adjacent tissue (Fig. 4). At this point, an increase in sugar content should play a role in providing nutrients for RKNs. The expression levels of many genes related to sucrose metabolism and transport also change after RKN infection [19, 20, 22, 23]. SUT, SWEET, SUS, invertase, INV-INH and SPS are thought to play important roles in plant growth, development, resistance to stress, etc [31]. . . The sucrose produced by plant photosynthesis is loaded into SEs/CCs and then reached the sink organs though phloem long distance transport. When the unloading pathway is an apoplastic pathway, the sucrose, and/or monosaccharide decomposed by SUS or INV was unloaded from SEs/CCs to the apoplastic space via SWEETs, then sucrose is either transported by SUTs to the sink cells, or sucrose is hydrolyzed to monosaccharides by cell wall acid invertase (CWINV), which are transported to the sink cells by STPs/HTs [14, 42]. SPS may resynthesize sucrose from monosaccharide derivatives [43].

Different sizes of galls were subjected to RNA-seq analysis in the present study, and 55 genes related to sugar metabolism and transport were found to be expressed in the galls. Among these genes, *SUT1*, *INV6*, *INV-INH1*, *INV-INH2*, *SWEET7a*, *STP1*, *STP2*, *STP10*, *STP12 and SUS3* were differentially expressed, suggesting that these genes may play key roles in sugar delivery to galls (Fig. 5; Supplementary Table S2). Further qRT‒PCR analysis of galls and adjacent tissue at 7 d and 14 d after RKN inoculation showed that the expression of *SUT1*, *SUT2*, *SWEET7a*, *SWEET17*, *STP1*, *STP2*, *STP10*, *STP12*, and *SUS3* was upregulated, while *INV-INH1*, *INV-INH2* and *SPS1* expression was downregulated in galls compared to uninoculated control roots (Fig. 6). Between galls and the adjacent tissue, the *SUT1*, *SUT2*, *SWEET7a*, *STP10*, *SUS3* and *SPS1* transcript levels in the galls were greater than those in the adjacent tissue, although the opposite was true for *STP1*, *STP2* and *STP12*. SlSWEET7a is considered to transport sucrose and hexose [40] and the activity of invertase can be inhibited by the inhibitor INV-INH [33]. These findings suggest that SUT1, SUT2, SWEET7a, STP10, SUS3 and SPS1 may provide sugar sources for GCs when STP1, STP2 and STP12 transport more sugar to phloem parenchyma cells for root development instead of for the gall development.

### Sugar delivery of tomato root at the early stage after RKNs inoculation

Many reports have shown that sugar levels in plants are significantly upregulated after RKN infection [21, 22, 44]. In tomato, a previous study showed that the levels of sucrose, fructose, glucose and ribose were upregulated in tomato stems after 2 months of infection with *M. incognita* [21], and another study indicated that soluble sugar (fructose, glucose, sucrose) content was upregulated in tomato leaves and roots during the early infestation stage, the function of which was to temporarily provide nutrients for RKNs [22]. However, we analyzed the soluble sugar levels in the tomato roots and leaves and found that the sucrose content in the roots and leaves gradually increased, but the glucose and fructose levels tended to decrease (Fig. 7A and B). Many studies have shown that in plants, changes in soluble sugar content are often involved as signals in plant responses to abiotic and biological stresses [45–50]. We also found that the important gene *SnRK1.1* in the sugar signaling pathway was significantly upregulated at the early stage after RKNs inoculation (Fig. 7F), suggesting that SnRK1.1 may be a key protein involved in sugar signal transduction after RKN infection. However, because sugar changes can also be involved in sucrose consumption and conversion, further studies need to be conducted to investigate a possible role for sugar signaling in defense against nematodes.

Changes in sugar levels must be inseparable from the roles of sugar metabolism and transport-related proteins. The expression levels of many sugar transporters, such as SUT, SWEET, the tonoplast monosaccharide transporter (TMT), and vacuolar glucose transporter (VGT) gene family members, were upregulated in Arabidopsis leaves or roots after RKN infection [22, 51]. In this study, the expression levels of 17 sugar metabolism- and transport-related genes, *SUT*, *SWEET*, *STP*, *INV*, *INV-INH*, *SUS* and *SPS*, were analyzed, and the results indicated that the expression levels of *SWEET7a*, *SPS1*, *INV-INH1* and *INV-INH2* were significantly upregulated, while *SUS1* and *SUS3* were significantly down regulated, suggesting that they were likely to contribute to the increase in sucrose content and the decrease in hexose content. Although the expression of the hexose transporter gene *STP10* was also upregulated, but the up-regulation occurred between 72 h and 96 h after RKN infection, and it was inconsistent with the decrease of glucose and fructose content in roots. Combined with the expression of *STP10* gene and sugar level changes in galls, we are more inclined to think that it played a role in the development of galls.

## Conclusion

**Fig. 8:**
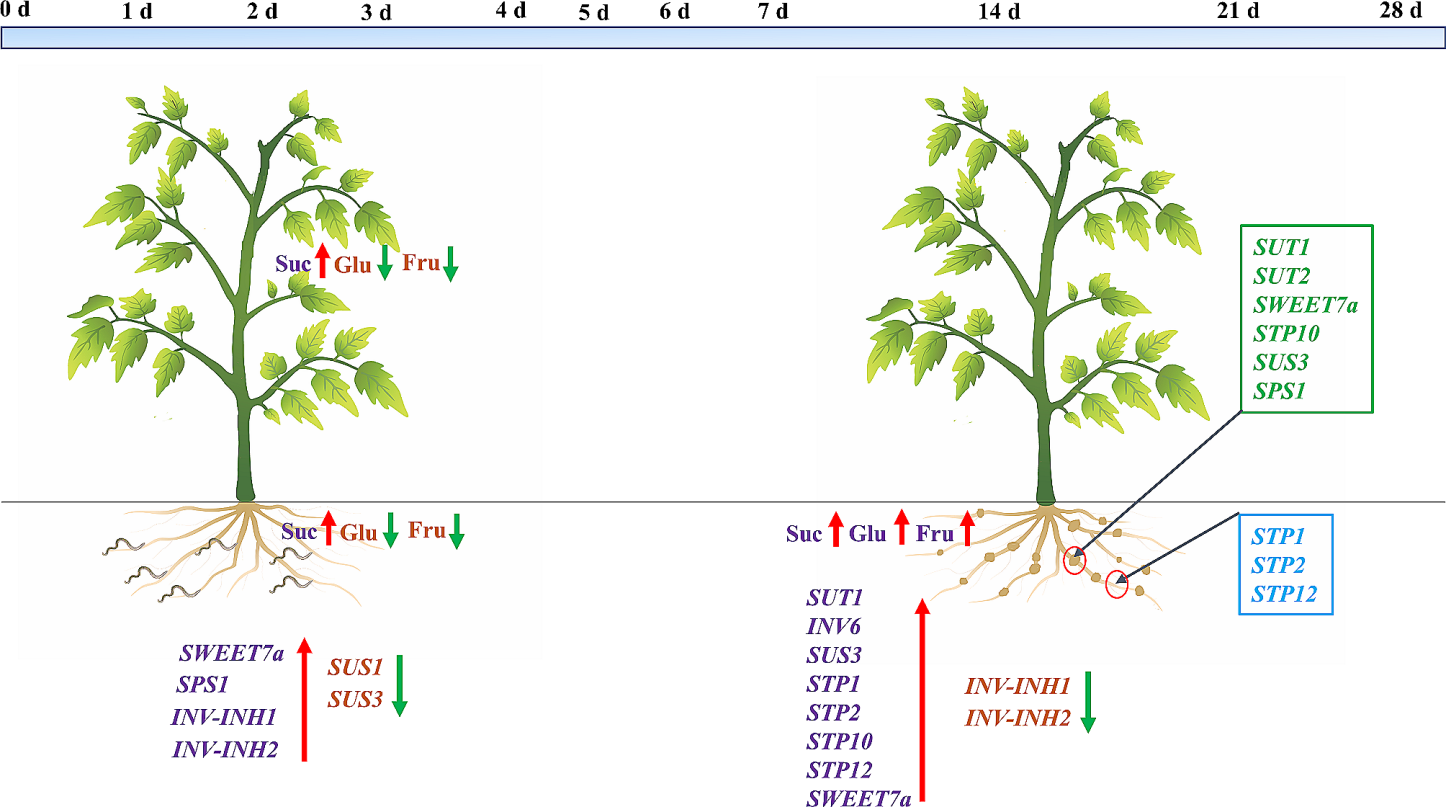
Model of changes in sugar content and response of genes related to sugar metabolism in tomato plants after *M. incognita* infestation. At the early stage of *M. incognita* infestation (1d − 4d), the sucrose content in tomato roots and leaves increased, while the glucose and fructose contents decreased. The up-regulation of gene expression levels of *SWEET7a*, *SPS1*, *INV-INH1* and *INV-INH2*, and the down-regulation of gene expression levels of *SUS1* and *SUS3* likely play key roles in the changes in sugar content, which correlated with their functions. With the development of the gall (7d − 28d), the contents of sucrose, glucose and fructose in the galls and adjacent tissue increase gradually, and the soluble sugars in phloem cells need to be transported to GCs through the apoplastic pathway. The up-regulation of *SUT1*, *INV6*, *SUS3*, *STP1*, *STP2*, *STP10*, *STP12*, *SWEET7a* and the down-regulation of *INV-INH1* and *INV-INH2* appear to be responsible for the rise in sugar levels. The expression levels of *SUT1*, *SUT2*, *SWEET7a*, *STP10*, *SUS3* and *SPS1* in the galls were greater than those in the adjacent tissue, suggesting that their proteins may provide sugar sources for GCs. However, the opposite results were obtained for *STP1*, *STP2* and *STP12*, suggesting that their proteins may transport more sugar to PPs for root development than for the gall development, which may be a defense measure for plants in response to RKNs invasion. Abbreviations: Suc, sucrose; Glu, glucose; Fru, fructose

Therefore, at the early stage of *M. incognita* infestation, the sucrose content in tomato roots and leaves increased, while the glucose and fructose contents decreased. *SWEET7a*, *SPS1*, *INV-INH1*, *INV-INH2*, *SUS1* and *SUS3* likely play key roles in root sugar delivery. Soluble sugars in phloem cells need to be transported to GCs through the apoplastic pathway. With the development of the gall, the contents of sucrose, glucose and fructose in the galls and adjacent tissue increase gradually, and *SUT1*, *INV6*, *SUS3*, *STP1*, *STP2*, *STP10*, *STP12*, *SWEET7a*, *INV-INH1* and *INV-INH2* appear to be responsible for the rise in sugar levels. *SUT1*, *SUT2*, *SWEET7a*, *STP10*, *SUS3* and *SPS1* may contribute more to the sugar supply of GCs, while *STP1*, *STP2* and *STP12* may transport more sugar to PPs for root development than for the gall development (Fig. 8).

In the future, we can try to inhibit or overexpress these genes related to sugar metabolism and transport, thereby altering the distribution of sugar in root galls and reducing sugar delivery to GCs. The insufficient sugar supply will cause RKNs to be stunted or unable to produce offspring, that may be an effective method for alleviating root-knot nematode disease.

### Electronic supplementary material

Below is the link to the electronic supplementary material.

**Supplementary Material 1**: **Supplementary fig. S1** Confocal laser scanning microscopy (CLSM) imaging of water unloading during the development of galls. **Supplementary table S1** List of gene ID and primers used in this study. **Supplementary table S2** FPKM value of these genes selected from RNA-sequence data.

## Data Availability

The raw sequencing data from this study have been deposited in the National Genomics Data Center (https://ngdc.cncb.ac.cn/), under the accession number: PRJCA016549.The accession numbers of all genes used in this paper were obtained from the Solanaceae Genomics Network (Version SL4.0 of the tomato genome, https://solgenomics.net/organism/Solanum_lycopersicum/genome). Accession number: *SlActin2 (Solyc11g005330); SlSTP1 (Solyc02g079220); SlSTP2 (Solyc09g075820); SlSTP10 (Solyc03g0066); SlSTP12 (Solyc05g018230); SlSUT1 (Solyc11g017010); SlSUT2 (Solyc05g007190); SlSWEET5b (Solyc06g071400); SlSWEET7a (Solyc08g082770); SlSWEET17 (Solyc01g099870); SlSPS1 (Solyc07g007790); SlSUS1 (Solyc07g042550); SlSUS3 (Solyc07g042550); SlINV3 (Solyc04g081440); SlINV-INH1 (Solyc12g099190); SlINV-INH2 (Solyc12g099200); SlHXK1 (Solyc03g121070); SlSnRK1.1 (Solyc02g067030); SlSnRK1.2 (Solyc03g115700)*.

## Abbreviations

RKN: Root-knot nematode
GC: Giant cells
SE: Sieve element
CC: Companion cell
PP: Phloem parenchyma cell
SWEET: Sugars Will Eventually be Exported Transporter
SUT: Sucrose transporter
SUS: Sucrose synthetase
STP: Sugar transport protein
INV: Invertase
INV-INH: Invertase inhibitor
SnRK: Sucrose non-fermenting-1-related protein kinase
HXK: Hexokinase
TMT: Tonoplast monosaccharide transporter
VGT: Vacuolar glucose transporter
G6PDH: Glucose-6-phosphate dehydrogenase
CF: Carboxyfluorescein
CFDA: Carboxyfluorescein diacetate
CLSM: Confocal laser scanning microscopy
HPLC: High-performance liquid chromatography
TEM: Transmission electron microscopy
FPKM: Fragments per kilobase of exon model per million mapped fragments
RNA-Seq: RNA sequencing
