# Supplementary material for: Sugar delivery at the tomato root and root galls after Meloidogyne incognita infestation
Source: BMC Plant Biol. 2024 May 24;24:451. doi: 10.1186/s12870-024-05157-7 (PMC11119304; doi:10.1186/s12870-024-05157-7)
Supplement: Supplementary file 1 — Supplementary Material 1: Supplementary fig. S1 Confocal laser scanning microscopy (CLSM) imaging of water unloading during the development of galls. Supplementary table S1 List of gene ID and primers used in this study. Supplementary table S2 FPKM value of these genes selected from RNA-sequence data. [file 12870_2024_5157_MOESM1_ESM.docx]

**Supplementary information**

**
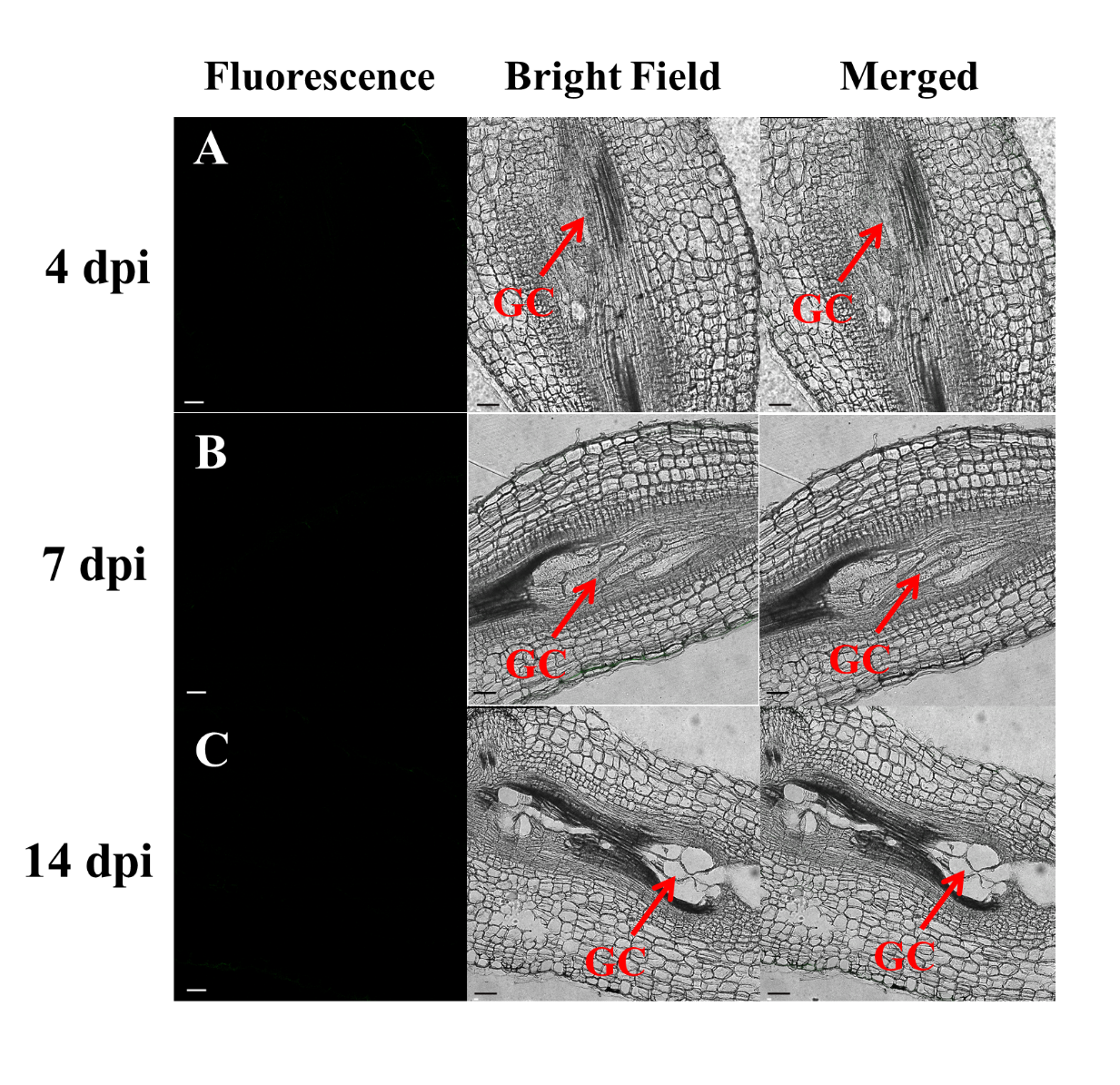
**

**Supplementary Fig. S1 Confocal laser scanning microscopy (CLSM) imaging of water unloading during the development of galls.** The root or gall treated with water instead of CFDA as tracing fluid were used as control and was sampled 24 h after treated, and was cut into 30 μm thickness slices using an oscillating microtome for fluorescence observation. **A-C**, Fluorescence images of root or gall after inoculation with RKN at 4d,7d and 14d, respectively. Scale Bars=20 μm. Abbreviation: GC, Giant cell.

**Table S1. List of gene ID and primers used in this study**

| Gene ID | Primer name | Primer sequence（5’ to 3’） |
| --- | --- | --- |
| Solyc11g005330 | SlActin2-qRT-F | TTGCTGACCGTATGAGCAAG |
|  | SlActin2-qRT-R | GGACAATGGATGGACCAGAC |
| Solyc02g079220 | SlSTP1-qRT-F | GGGCTACACAGTTGCTTTGC |
|  | SlSTP1-qRT-R | TGTTGTAGTGGGGATGATATTGAT |
| Solyc09g075820 | SlSTP2-qRT-F | GGAGATGACGGAGAGGGTGT |
|  | SlSTP2-qRT-R | GAGAAACACTTATCGATATCAC |
| Solyc07g006970 | SlSTP3-qRT-F | GTTCTTGCTGCATTTACCTCG |
|  | SlSTP3-qRT-R | AAGCATGCCAAGGTTGAC |
| Solyc03g006650 | SlSTP10-qRT-F | GCTGGTGGTGGTGGAATTAGT |
|  | SlSTP10-qRT-R | GGTTTGTTGATGTGTCTGCCTTT |
| Solyc05g018230 | SlSTP12-qRT-F | GGCTTGATATTCATCATGA |
|  | SlSTP12-qRT-R | CATTCCTGAAGGAATCCATTGTG |
| Solyc03g093410 | SlSTP16-qRT-F | GACAAGGAATTTGCCTATTG |
|  | SlSTP16-qRT-R | GGTGCCATTACTATTGAGTAC |
| Solyc11g017010 | SlSUT1-qRT-F | CATTTTTCAAGGCCGTCGG |
|  | SlSUT1-qRT-R | TCCGGGAGTTCGTTTTCAC |
| Solyc05g007190 | SlSUT2-qRT-F | GAATCCGACGCCGTTTCATA |
|  | SlSUT2-qRT-R | GATTAGAATCATTCATTTCTC |
| Solyc06g071400 | SlSWEET5b-qRT-F | CGGATTTGGTCTTGCTATTGAG |
|  | SlSWEET5b-qRT-R | TGAAGACTATAGCCACAATCCC |
| Solyc08g082770 | SlSWEET7a-qRT-F | CATCTTGTTCTTGTCACCCTTG |
|  | SlSWEET7a-qRT-R | CCCAAAGCCCACAATTGATAAA |
| Solyc05g024260 | SlSWEET12c-qRT-F | AGTCTCCATCAATTCCTTTGGT  AATCCAACCAACAACTTGTACG |
|  | SlSWEET12c-qRT-R | AATCCAACCAACAACTTGTACG |
| Solyc01g099870 | SlSWEET17-qRT-F | ATCTACTGTCAATGGCTTTGGA |
|  | SlSWEET17-qRT-R | CCATATAAAATGAACTGCGCCA |
| Solyc07g007790 | SlSPS1-qRT-F | GATAAACAACTGTGGGCGAAAT |
|  | SlSPS1-qRT-R | GAACTTCGGGGTCTAATGTACT |
| Solyc07g042550 | SlSUS1-qRT-F | TCATGGCATTAATGTGTTCGAC |
|  | SlSUS1-qRT-R | TCATTCTCAACGTCACTGTACA |
| Solyc07g042550 | SlSUS3-qRT-F | TTCTCGAGTTTCTTCGAGTTCA |
|  | SlSUS3-qRT-R | ATCATCTCTAGAACACGCTCTG |
| Solyc04g081440 | SlINV3-qRT-F | GAGAATTGTAACAGGATGCGAC |
|  | SlINV3-qRT-R | AATAAATCGACCAAGCTTTCCG |
| Solyc12g099190 | SlINV-INH1-qRT-F | TAGAGAAATCGAACCCTGAGTG |
|  | SlINV-INH1-qRT-R | CTTCAGCAAATTTAGGGACACC |
| Solyc12g099200 | SlINV-INH2-qRT-F | CTAATCCTCCTCAAGCTTGGAA |
|  | SlINV-INH2-qRT-R | CAACCATTCCATCTTCTGCAAA |
| Solyc03g121070 | SlHXK1-qRT-F | GAACTCTTTGAAGGACTTGCTG |
|  | SlHXK1-qRT-R | AAGGTACATTGAATGAGAGGCA |
| Solyc02g067030 | SlSnRK1.1-qRT-F | AACATCAAAGGAAAATGGACGG |
|  | SlSnRK1.1-qRT-R | CTCTATGACCTCATAAAGCCGT |
| Solyc03g115700 | SlSnRK1.2-qRT-F | TAGGAATATGGTGGTTCATCGG |
|  | SlSnRK1.2-qRT-R | GGACTTCCACAACTAGTCTTCA |

**Table S2. FPKM value of these genes selected from RNA-sequence data**

|  | **Gene name** | **Gene ID** | **CK_**  **FPKM** | **RK1_**  **FPKM** | **RK2_**  **FPKM** | **RK3_**  **FPKM** | **RK4_**  **FPKM** | **RK5_**  **FPKM** | **RK6_**  **FPKM** | **RK7_**  **FPKM** |
| --- | --- | --- | --- | --- | --- | --- | --- | --- | --- | --- |
| 1 | **SUT1** | **Solyc11g017010.1** | 203.40 | 308.90 | 221.13 | 256.96 | 279.90 | 279.57 | 235.15 | 212.99 |
| 2 | **SUT2** | **Solyc05g007190.2** | 22.57 | 24.39 | 25.65 | 23.84 | 27.03 | 25.92 | 24.28 | 23.55 |
| 3 | **SUT4** | **Solyc04g076960.2** | 19.79 | 12.24 | 25.98 | 29.00 | 17.28 | 19.18 | 16.05 | 17.48 |
| 4 | **INV1** | **Solyc10g083290.1** | 16.38 | 10.55 | 7.52 | 6.91 | 8.93 | 8.41 | 5.74 | 5.29 |
| 5 | **INV2** | **Solyc03g083910.2** | 27.73 | 0.90 | 0.20 | 0.23 | 0.14 | 0.34 | 0.72 | 0.66 |
| 6 | **INV3** | **Solyc04g081440.2** | 158.73 | 138.05 | 77.10 | 76.38 | 82.63 | 77.16 | 71.38 | 69.92 |
| 7 | **INV4** | **Solyc03g121680.1** | 0.09 | 0.02 | 0.49 | 0.16 | 0.02 | 0.14 | 0.38 | 0.07 |
| 8 | **INV 5** | **Solyc09g010080.2** | 0.02 | 0.02 | 0.02 | 0.02 | 0.02 | 0.02 | 0.02 | 0.02 |
| 9 | **INV 6** | **Solyc08g079080.2** | 6.44 | 71.09 | 11.85 | 13.18 | 45.58 | 39.10 | 37.20 | 35.95 |
| 10 | **INV 7** | **Solyc10g083300.1** | 3.26 | 3.84 | 0.06 | 0.02 | 0.06 | 0.10 | 0.02 | 0.06 |
| 11 | **INV 8** | **Solyc09g010090.2** | 4.85 | 5.29 | 2.41 | 2.94 | 1.62 | 2.94 | 2.76 | 2.99 |
| 12 | **INV-INH 3** | **Solyc01g088590.2** | 18.09 | 5.36 | 7.07 | 6.67 | 17.53 | 11.24 | 9.69 | 16.97 |
| 13 | **INV-INH 1** | **Solyc12g099190.1** | 236.96 | 52.76 | 40.71 | 65.59 | 67.46 | 99.70 | 91.48 | 140.42 |
| 14 | **INV-INH 2** | **Solyc12g099200.1** | 261.89 | 100.07 | 67.69 | 64.44 | 83.67 | 97.78 | 99.98 | 112.01 |
| 15 | **SUS4** | **Solyc09g098590.2** | 21.44 | 2.44 | 4.14 | 5.55 | 5.13 | 5.15 | 8.79 | 10.42 |
| 16 | **SUS7** | **Solyc02g081300.2** | 14.43 | 8.59 | 11.82 | 17.40 | 4.22 | 3.20 | 4.69 | 2.73 |
| 17 | **SUS3** | **Solyc07g042550.2** | 342.58 | 289.54 | 374.31 | 691.06 | 733.58 | 886.43 | 847.39 | 933.23 |
| 18 | **SUS4** | **Solyc07g042520.2** | 261.58 | 28.09 | 239.83 | 270.61 | 182.60 | 157.10 | 275.53 | 271.44 |
| 19 | **STP1** | **Solyc02g079220.2** | 72.95 | 586.37 | 388.97 | 348.83 | 681.30 | 602.77 | 570.00 | 611.24 |
| 20 | **STP2** | **Solyc09g075820.2** | 11.68 | 46.23 | 45.52 | 43.99 | 58.44 | 41.68 | 46.90 | 57.84 |
| 21 | **STP3** | **Solyc07g006970.2** | 4.93 | 10.16 | 2.13 | 2.74 | 4.28 | 4.25 | 3.10 | 4.17 |
| 22 | **STP7** | **Solyc03g005140.1** | 0.35 | 1.38 | 0.92 | 0.61 | 0.49 | 0.38 | 0.29 | 0.62 |
| 23 | **STP10** | **Solyc03g006650.1** | 0.04 | 43.90 | 5.83 | 2.79 | 6.26 | 11.10 | 2.68 | 3.01 |
| 24 | **STP11** | **Solyc06g054270.2** | 0.36 | 0.80 | 0.69 | 0.60 | 0.91 | 1.38 | 0.80 | 0.82 |
| 25 | **STP12** | **Solyc05g018230.2** | 8.11 | 48.70 | 18.75 | 24.27 | 42.65 | 38.45 | 29.48 | 33.56 |
| 26 | **STP15** | **Solyc03g093400.2** | 3.67 | 0.54 | 0.98 | 1.14 | 2.18 | 2.20 | 2.02 | 1.73 |
| 27 | **STP16** | **Solyc03g093410.2** | 26.60 | 23.03 | 1.87 | 2.10 | 12.89 | 9.19 | 5.86 | 5.52 |
| 28 | **STP17** | **Solyc03g094170.1** | 3.54 | 0.02 | 1.30 | 0.02 | 0.02 | 0.02 | 0.02 | 1.12 |
| 29 | **NEC1** | **Solyc09g074530.2** | 0.29 | 0.00 | 0.00 | 0.00 | 0.00 | 0.30 | 0.24 | 0.76 |
| 30 | **SWEET1b** | **Solyc04g064620.2** | 32.19 | 9.44 | 7.66 | 7.52 | 9.40 | 9.89 | 8.87 | 15.16 |
| 31 | **SWEET1c** | **Solyc04g064630.2** | 218.39 | 30.89 | 7.73 | 4.97 | 9.80 | 8.38 | 5.43 | 16.77 |
| 32 | **SWEET1d** | **Solyc04g064640.2** | 3.02 | 0.98 | 1.59 | 2.23 | 1.11 | 1.60 | 1.52 | 0.87 |
| 33 | **SWEET1e** | **Solyc06g060590.2** | 25.70 | 13.75 | 10.62 | 7.81 | 5.53 | 4.62 | 11.22 | 12.21 |
| 34 | **SWEET1f** | **Solyc06g060580.1** | 2.02 | 0.39 | 0.62 | 0.23 | 0.19 | 0.00 | 0.04 | 0.12 |
| 35 | **SWEET2b** | **Solyc07g062120.2** | 7.74 | 3.72 | 2.60 | 2.22 | 3.77 | 4.34 | 4.25 | 5.24 |
| 36 | **SWEET3** | **Solyc03g007360.2** | 18.81 | 1.12 | 1.15 | 0.19 | 0.27 | 0.48 | 0.62 | 0.34 |
| 37 | **SWEET5b** | **Solyc06g071400.2** | 1.94 | 5.20 | 5.70 | 4.21 | 3.71 | 3.13 | 2.54 | 3.36 |
| 38 | **SWEET7a** | **Solyc08g082770.2** | 0.00 | 1.15 | 16.46 | 11.29 | 13.20 | 9.61 | 4.82 | 7.28 |
| 39 | **SWEET10a** | **Solyc03g097580.2** | 11.15 | 3.59 | 0.24 | 0.07 | 0.32 | 0.36 | 0.15 | 0.57 |
| 40 | **SWEET10b** | **Solyc03g097600.2** | 17.38 | 9.55 | 2.99 | 3.22 | 2.84 | 3.37 | 3.19 | 2.35 |
| 41 | **SWEET10c** | **Solyc03g097610.2** | 2.07 | 1.45 | 0.48 | 0.30 | 0.50 | 0.52 | 0.52 | 0.50 |
| 42 | **SWEET11b** | **Solyc03g097570.2** | 14.58 | 2.66 | 0.26 | 0.10 | 0.03 | 0.43 | 0.24 | 0.47 |
| 43 | **SWEET11c** | **Solyc06g072620.2** | 3.61 | 2.56 | 0.70 | 1.03 | 1.65 | 1.34 | 0.71 | 0.69 |
| 44 | **SWEET12a** | **Solyc03g097590.2** | 3.88 | 1.43 | 0.58 | 1.07 | 0.63 | 0.61 | 1.57 | 0.98 |
| 45 | **SWEET12b** | **Solyc03g097620.1** | 2.07 | 0.60 | 0.00 | 0.00 | 0.00 | 0.00 | 0.00 | 0.00 |
| 46 | **SWEET12c** | **Solyc05g024260.2** | 2.53 | 1.81 | 0.25 | 0.00 | 2.73 | 4.57 | 3.01 | 2.34 |
| 47 | **SWEET12d** | **Solyc06g072630.2** | 2.39 | 5.98 | 0.56 | 0.73 | 0.22 | 0.32 | 0.12 | 0.00 |
| 48 | **SWEET14** | **Solyc03g097560.2** | 0.00 | 0.59 | 0.00 | 0.00 | 0.50 | 0.14 | 0.15 | 0.00 |
| 49 | **SWEET16** | **Solyc01g099880.2** | 17.03 | 0.30 | 0.85 | 0.95 | 2.10 | 1.69 | 3.52 | 11.17 |
| 50 | **SWEET17** | **Solyc01g099870.1** | 1.05 | 4.69 | 2.54 | 1.83 | 5.13 | 0.42 | 2.12 | 0.60 |
| 51 | **SPS1** | **Solyc07g007790.2** | 83.47 | 5.96 | 28.10 | 43.14 | 25.81 | 18.29 | 21.26 | 17.22 |
| 52 | **SPS2** | **Solyc08g042000.2** | 2.04 | 1.02 | 1.21 | 2.18 | 0.88 | 0.62 | 0.81 | 0.49 |
| 53 | **SPS3** | **Solyc09g092130.2** | 0.12 | 0.49 | 0.44 | 1.15 | 0.32 | 0.21 | 0.17 | 0.15 |
| 54 | **SPS4** | **Solyc11g045100.1** | 6.21 | 4.66 | 7.93 | 7.85 | 3.48 | 3.34 | 2.97 | 2.81 |
| 55 | **SPS5** | **Solyc11g045110.1** | 7.08 | 0.45 | 0.42 | 0.97 | 0.48 | 0.35 | 0.81 | 0.58 |
